# Supplementary material for: WordCommentsAnalyzer: A windows software tool for qualitative research
Source: F1000Res. 2018 Sep 4;7:536. Originally published 2018 May 3. [Version 2] doi: 10.12688/f1000research.14819.2 (PMC6137411; doi:10.12688/f1000research.14819.2)
Supplement: Supplementary file 3 [file f1000research-7-17614-s0002.tgz › b2a79496-4ada-47c4-9682-a99ce867836d.zip!/Eight Iranian Health Journals/Journal of Health System Research _31.docx]

© Journal of Health System Research . This work is licensed under CC BY-NC 4.0 (https://creativecommons.org/licenses/by-nc/4.0/).

Volume & Issue: دوره 9، شماره 1؛ 1392

Title: با سرطان پروستات PUFA بررسی ارتباط اسیدهای چرب غیر اشباع با چند پیوند دوگانه

http://hsr.mui.ac.ir/index.php/jhsr/article/view/639

از شایع‌ترین سرطان‌های شناخته شده در امریکا، کانادا و ششمین سرطان شناخته شده در جهان، سرطان پروستات می‌باشد. فاکتورهای غذایی نقش مهمی در پیشرفت سرطان پروستات دارد. یکی از آن‌ها، دریافت چربی‌ها به ویژه دریافت اسیدهای چرب غیر اشباع با چند پیوند دوگانه (Polyunsaturated fatty acid یا PUFA) می‌باشد. از این رو هدف از این بررسی، مروری بر پژوهش‌های انجام شده در زمینه ارتباط دریافت PUFA با سرطان پروستات بود. با جستجو در PubMed مقالات با طراحی‌های مورد شاهدی، کوهورت، کوهورت آینده‌نگر و آینده‌نگر انتخاب شد. بنابر نتایج مطالعه‌های حیوانی، ارتباط مستقیم بین اسیدهای چرب امگا 6 با رشد تومور و ارتباط معکوس بین اسیدهای چرب امگا 3 با رشد تومور پروستات مشاهده گردید. اسیدهای چرب امگا 3 سوبسترا برای تولید ایکوزانوئیدهای ضد التهابی هستند. در مقابل اسیدهای چرب امگا 6 سوبسترا برای تولید ایکوزانوئیدهای پیش التهابی می‌باشند. در اکثر مطالعه‌های انسانی ارتباط معکوس بین مصرف ماهی با سرطان پروستات و ارتباط مستقیم بین آلفا لینولنیک اسید با سرطان پروستات مشاهده گردید. مطالعه‌های انسانی نتایج متفاوتی در زمینه ارتباط PUFA با سرطان پروستات بیان کردند. اسیدهای چرب امگا 3 از منابع دریایی و گیاهی اثرات متفاوتی روی سرطان پروستات داشتند. در اکثر مطالعه‌ها آلفا لینولنیک اسید مرتبط با افزایش خطر سرطان پروستات بود. از این رو نیاز به مطالعه‌های بیشتر در زمینه ارتباط PUFA با سرطان پروستات می‌باشد.

Title: اخلاق در آموزش بهداشت: آيا آموزش‌دهندگان بهداشت با آن آشنا هستند؟

http://hsr.mui.ac.ir/index.php/jhsr/article/view/652

به دلیل خطرات و عدم اطمینان‌ها در ارتباط با تحقیق‌ها و مداخلات پزشکی، ملاحظات اخلاقی اغلب برای تحقیق‌های پزشکی ضروری می‌باشند. متأسفانه در حوزه آموزش بهداشت و ارتقای سلامت به این ملاحظات اخلاقی به طور معمول پرداخته نشده است. در ایران منابع و متون مربوط به اخلاق و آموزش بهداشت بسیار محدود است و اميد مي‌رود که مقاله حاضر گامی کوچک در راستای پر کردن گوشه‌ای از این فقدان بزرگ و جدی باشد. این مقاله به صورت مروری (Review article) به منابع و متون مربوط به اخلاق در حرفه تخصصی آموزش بهداشت پرداخته است. در این مقاله ابتدا کلیاتی در خصوص اخلاق، منشأ پیدایش آن، رویکردها و نظریه‌های حاکم در خصوص اخلاق آورده شده است. پس از آن در بخش ديگري برای اولین بار نسخه فارسی مربوط به کدهای اخلاقی حرفه آموزش بهداشت (در 6 بند و 47 بخش) آورده شده است که توسط (CNHEO) Coalition of national health education organizations تصویب گردیده است. به طور كلي يكی از عمده‌ترین مسؤوليت‌های آموزش‌‌دهندگان بهداشت کمک به افراد در جهت اتخاذ تصمیمات عاقلانه مرتبط با سلامتشان می‌باشد. آموزش‌دهندگان بهداشت در عملکرد حرفه‌ای خود با طیفی از موقعیت‌های دشوار و بلاتکلیفی‌ها مواجه می‌شوند. از طرف دیگر آموزش‌دهندگان بهداشت و ارتقادهندگان سلامت خواسته يا ناخواسته به صورت یک الگوی نقش (Role model) برای دیگران عمل می‌کنند. نویسندگان این مقاله آموزش‌های تخصصی و کاربردی را در خصوص موضوعات اخلاقی در آموزش بهداشت برای متخصصان، پژوهشگران، دانشجویان و سایر فعالان در حوزه آموزش بهداشت و ارتقای سلامت توصیه می‌کنند.

Title: ارتباط دريافت اسيدهاي چرب چند غير اشباع (PUFA)، چاقي و چاقي شکمي در دختران دانشجوي دانشگاه علوم پزشکي اصفهان

http://hsr.mui.ac.ir/index.php/jhsr/article/view/279

مقدمه: در حال حاضر، چاقي به يک مشکل جهاني تبديل شده است. مطالعه‌های زيادي در مورد چاقي و چاقي شکمي انجام شده است. از این رو در اين تحقيق ارتباط دريافت Poly unsaturated fatty acid (PUFA) با شيوع چاقي و چاقي شکمي در دختران دانشجوي دانشگاه علوم پزشکي اصفهان مورد بررسی قرار گرفت.  روش‌ها: اين تحقيق روي 160 نفر دانشجوي دختر 30-18 سال و به روش مقطعي انجام شد. دانشجويان به روش تصادفي انتخاب شدند. از افراد فرم بسامد خوراك نيمه کمي اعتبارسنجي شده گرفته شد. فعالیت بدنی با استفاده از پرسش‌نامه استاندارد فعالیت بدنی ارزیابی شد. جهت بررسي ارتباط PUFA با شاخص‌هاي آنتروپومتريک از Linear regression، Logistic regression و آزمون همبستگی استفاده شد. آنالیزها با استفاده از نرم‌افزار SPSS (SPSS Inc., Chicago, IL) انجام شد. یافته‌ها: نتايج اين مطالعه حاکي از آن بود که شيوع چاقي 5/2 درصد، چاقي شکمي 3/1 درصد و اضافه وزن 4/9 درصد است. ميانگين وزن، Body mass index (BMI) و دور کمر به ترتيب برابر 21/56 کيلوگرم، 88/21 کيلوگرم بر متر مربع و 94/70 سانتي‌متر بود. همچنين ميانگين دريافت چربي و PUFA به ترتيب 11/74 و 54/17 گرم در روز بود. نتیجه‌گیری: ارتباط معني‌داري بين مصرف PUFA با وزن، دور کمر، اضافه وزن، چاقي و چاقي شکمي در دانشجويان دختر مشاهده نشد (05/0 P >).

Title: مصرف سیب‌زمینی و خطر سکته مغزی: یک مطالعه مورد- شاهدی

http://hsr.mui.ac.ir/index.php/jhsr/article/view/557

مقدمه: مطالعه‌های قبلی در ارتباط با دریافت‌های غذایی سیب‌زمینی و سکته مغزی، بیشتر روی نمایه گلایسمیک متمرکز بودند و اطلاعاتی چندانی در مورد رابطه مصرف سیب‌زمینی و خطر سکته مغزی عنوان نمی‌کردند. هدف از این مطالعه ارزیابی ارتباط میان مصرف سیب‌زمینی و خطر سکته مغزی در جامعه بزرگسال ایرانی بود.  روش‌ها: 195 بیمار مبتلا به سکته بستری در بخش نورولوژی بیمارستان الزهرا (س) و 195 فرد شاهد از سایر بخش‌ها با نمونه‌گیری آسان غیر تصادفی انتخاب و در دو گروه از نظر جنس و سن همسان شدند. دریافت‌های غذایی شرکت‌کنندگان از طریق پرسش‌نامه بسامد خوراک جمع‌آوری شد. اطلاعات دیگر توسط پرسش‌نامه جمع‌آوری شد. یافته‌ها: میانگین مصرف سیب‌زمینی در دو گروه مورد و شاهد به ترتیب 4/3 ± 1/31 و 3/1 ± 4/23 بود. مصرف بیشتر سیب‌زمینی در افرادی که جوان‌تر بودند و فعالیت فیزیکی بیشتری داشتند، مشاهده شد. مصرف بالاتر سیب‌زمینی با دریافت بیشتر انرژی، میوه‌ها، سبزیجات، حبوبات و غلات در ارتباط بود. پس از تعدیل از نظر سن، جنس و کل انرژی دریافتی، افرادی که مصرف سیب‌زمینی بیشتری داشتند، بیشتر در معرض ابتلا به سکته مغزی بودند (9/1 OR:؛ 6/3 و 0/1 CI:). حتی پس از تعدیل بیشتر برای استعمال سیگار و میزان فعالیت فیزیکی ارتباط همچنان معنی‌دار بود (9/1 :OR؛ 6/3 و 0/1 :CI). تعدیل بیشتر برای دریافت‌های غذایی موجب شد تا ارتباط از حالت معنی‌دار خارج شود (1/1 :OR؛ 5/2 و 5/0 :CI). با این وجود با در نظر گرفتن شاخص توده بدنی در مدل نهایی مشخص شد که افرادی که در چارک سوم مصرف سیب‌زمینی قرار داشتند، به طور معنی‌داری بیشتر در معرض خطر سکته مغزی بودند (2/2 :OR؛ 7/4 و 0/1 :CI). نتیجه‌گیری: ارتباط مستقل معنی‌دار مرزی میان مصرف سیب‌زمینی و خطر سکته وجود داشت. جهت تأیید این یافته، مطالعه‌های بیشتری نیاز است.

Title: بانک اطلاعاتی ظرفیت هوازی (VO2-max) بر اساس سن کارگران مرد شاغل در بخش صنعت شهرستان شیراز

http://hsr.mui.ac.ir/index.php/jhsr/article/view/633

مقدمه: برای ایجاد تناسب فیزیولوژیک بین کار و شاغلان و همچنین انتخاب افراد مناسب برای انجام کارهای مختلف می‌توان از پارامترهای گوناگون از جمله بیشترین ظرفیت هوازی (VO2-max) بهره جست. این مطالعه با هدف ایجاد بانک اطلاعاتی برآورد VO2-max در کارگران مرد بخش صنعت صورت گرفت.  روش‌ها: در این مطالعه مقطعی به تعداد 500 نفر از کارگران مرد کارخانجات مختلف شهر شيراز با گستره سنی20 تا 59 سال که از سلامتی برخوردار بودند، داوطلبانه شرکت نمودند. این کارگران به وسيله دوچرخه ارگومتر بر اساس پروتکل آستراند به مدت 6 دقیقه مورد ارزيابی قرار گرفتند. ابزار جمع‌آوری داده‌ها، پرسش‌نامه ویژگی‌های فردی بود که خود از دو قسمت تشکیل شده بود. بخش اول ویژگی‌های دموگرافیک و بخش دوم ویژگی‌های آنترپومتریک و فیزیولوژیک فرد را شامل می‌شد. یافته‌ها: صدک پنجم، پنجاهم و نود و پنجم بیشترین ظرفیت هوازی در کارگران مورد مطالعه به ترتیب برابر با 07/2، 72/2 و 02/3 لیتر در دقیقه برآورد شد. نتایج این مطالعه نشان داد که بیشترین ظرفیت هوازی جامعه مورد مطالعه به ازای هر دهه تقریباً به میزان 10 درصد کاهش می‌یابد. نتیجه‌گیری: وجود چنین بانک اطلاعاتی می‌تواند به استخدام افراد در کارهای مناسب و همچنین ارزیابی تناسب فیزیولوژیک کار با شاغلان کمک شایان توجهی کند.

Title: عوامل مؤثر بر استرس شغلی پرستاران شاغل در بخش فوریت‌های پزشکی

http://hsr.mui.ac.ir/index.php/jhsr/article/view/638

مقدمه: پرستاری یکی از حرفه های پر استرس است. عوامل استرس زای فراوان موجود در این حرفه تاثیرات زیادی را در کارکنان آن به جای می گذارد.  این مطالعه با هدف شناسایی علل ایجاد کننده استرس شغلی در پرستاران بخش فوریت ها پزشکی انجام شده است.   روش تحقیق: در این مطالعه مقطعی 84 نفر از کارکنان شاغل در بیمارستان در دو گروه پرستاران زن شاغل در بخش فوريت هاي پزشكي و کارکنان زن شاغل در بخش های مختلف اداری بیمارستان انتخاب شدند (42 نفر در هر گروه). ابزار جمع آوری اطلاعات نسخه فارسی و خلاصه پرسشنامه استرس شغلی سازمان بین المللی ایمنی و سلامت کار بود. پس از جمع آوری داده ها، به منظور تجزیه و تحلیل اطلاعات و از آزمون های آزمون هاي آماری تی مستقل، تحلیل واریانس و همبستگی پیرسون استفاده شد.   یافته ها: میانگین نمره استرس شغلی در پرستاران 29/9±30/97 و در کارمندان 91/10±85/91 بود (016/0=P). نتایج اختلاف معناداری بین دو گروه مورد مطالعه در میانگین نمره بار نقش (005/0=P)، کنترل نقش(02/0=P) و حمایت اجتماعی (004/0=P) نشان می دهد.   نتیجه گیری: حاضر پرستاران بخش فوریت های پزشکی استرس شغلی بالایی را در مقایسه با کارمندان بیمارستان تجربه می نمایند. ضمن آنکه  بار نقش، کنترل نقش و حمایت اجتماعی به عنوان مهمترین عوامل در استرس شغلی پرستاران مطرح می باشند.

Title: بررسی شیوع استرس شغلی و ارتباط آن با ویژگی‌های فردی در کارگران یک صنعت تونل‌سازی

http://hsr.mui.ac.ir/index.php/jhsr/article/view/651

مقدمه: استرس شغلی امروزه یکی از پدیده‌های مهم در زندگی اجتماعی و تهدیدی جدی برای سلامتی نیروی کار در جهان می‌باشد. این مطالعه با هدف بررسی میزان شیوع استرس شغلی و تعیین ارتباط آن با ویژگی‌های فردی کارگران در یکی از صنایع انجام شد.  روش‌ها: این مطالعه توصیفی تحلیلی به صورت مقطعی در سال 1390 روی 145 نفر از مجموع 450 نفر از کارکنان شاغل در یک پروژه تونل‌سازی واقع در شهرستان کرج انجام شد. جهت تعیین استرس شغلی از پرسش‌نامه استاندارد استرس شغلی مؤسسه Health and safety executive (HSE) انگلستان استفاده گردید. تجزیه و تحلیل داده‌ها با استفاده از نرم‌افزار SPSS نسخه 16 و آزمون‌های آماری 2c، Independent t و تحلیل One Way ANOVA صورت گرفت. یافته‌ها: از نظر آماری ارتباط معنی‌داری بین استرس شغلی با متغیرهای ورزش، تحصیلات مرتبط با شغل و سابقه شغلی وجود داشت (05/0 > P)؛ به طوری که افراد دارای تحصیلات مرتبط شغلی، بدون سابقه شغلی و افرادی که عادت به ورزش کردن داشتند، میانگین استرس شغلی کمتری داشتند. همچنین بین استرس شغلی با متغیرهای سن، تأهل، سطح تحصیلات، مصرف دخانیات، بیماری‌های مزمن و شاخص توده بدنی ارتباط آماری معنی‌داری دیده نشد (05/0 < P). نتیجه‌گیری: توجه به استرس شغلی در صنایع ساختمانی ضروری است. اقدامات کنترلی نظیر آموزش کارگران و طراحی و اجرای برنامه‌های ورزشی در محیط‌های شغلی می‌تواند در کاهش میزان استرس شغلی مؤثر واقع گردد.

Title: شیوع آنمی فقر آهن در زنان باردار با بارداری‌های ناخواسته و پرخطر در استان اصفهان و عوامل مؤثر بر آن

http://hsr.mui.ac.ir/index.php/jhsr/article/view/816

مقدمه: این مطالعه با هدف بررسی شیوع آنمی در زنان باردار با بارداری‌های ناخواسته و پرخطر و عوامل مؤثر بر آن در استان اصفهان انجام شد.  روش‌ها: این مطالعه توصیفی تحلیلی بود. 7233 مادر باردار متناسب با تعداد زنان باردار هر شهرستان و بر حسب مناطق شهری و روستایی با نمونه‌گيري تصادفي تسهیم به سهم شد. ميزان هموگلوبين و ساير اطلاعات با استفاده از فرم ثبت اطلاعات از طريق مراجعه به پرونده خانوار مادر باردار جمع‌آوري گرديد. داده‌ها با استفاده از نرم‌افزار SPSS نسخه 5/11 با استفاده از آمار توصيفي استنباطي تجزیه و تحلیل گرديد. یافته‌ها: شيوع آنمي در استان 4/9 درصد بود كه دامنه آن بین 2/27-6/2 در شهرستان‌ها متغير بود. اين شيوع در شهرستان‌هاي تابعه استان از نظر آماري معني‌دار بود (001/0 < P)، ولي بين شيوع آنمي در مناطق شهري و روستايي تفاوت آماري معني‌داري مشاهده نشد. بین شیوع آنمی و گروه‌های سنی (006/0 = P)، تعداد باروری (022/0 = P)، مراقبت قبل از بارداری (016/0 = P) و سن بارداری (001/0 > P) و بین بارداری ناخواسته و سن مادر (001/0 > P) تفاوت آماری معنی‌داری وجود داشت. ارتباط معكوس و معني‌داري بين شيوع آنمي با تحصيلات مادر مشاهده گرديد (001/0 < P). نتایج آنالیز رگرسیون نشان داد که سن بارداری، سن مادر، ناخواسته بودن بارداری، فواصل بارداری و تحصیلات مادر اثر معنی‌داری بر ابتلا به آنمی داشت. نتیجه‌گیری: نتايج اين مطالعه ضرورت توجه به گسترش مراقبت‌هاي قبل از بارداري در زمينه الگوي صحيح غذايي و مصرف منظم مكمل آهن، ترويج برنامه مكمل ياري آهن و اسيد فوليك در دوران قبل، حين و پس از بارداري و همچنين پيشگيري از بارداري در سنين بالا و بارداري‌هاي ناخواسته را نمايان مي‌سازد.

Title: بررسی اشتباهات دارویی و علل عدم گزارش آن از دیدگاه پرستاران بیمارستان‌های دانشگاه ‌علوم پزشکی اصفهان

http://hsr.mui.ac.ir/index.php/jhsr/article/view/877

مقدمه: خطاهای‌ دارویی به‌ عنوان یک ‌مشکل‌ جهانی می‌تواند منجر به صدمات‌ جدی و حتی مرگ بیماران‌ شود. نتیجه ‌اولیه و طبیعی اشتباهات ‌دارویی، افزایش ‌مدت بستری ‌بیمار در بیمارستان و نیز افزایش ‌سرسام‌آور هزینه‌ها خواهد بود. به‌ طور کلی خطاهای‌ دارویی روی ‌بیماران، پرستاران‌ و سازمان‌ها تأثیرات منفی دارد و منجر به‌ کاهش‌ کیفیت مراقبت‌های ارایه‌ شده ‌می‌گردد. هدف از‌ این مطالعه بررسی ‌میزان بروز خطاهای ‌دارویی و تعیین علل گزارش ‌نکردن این اشتباهات در پرستاران بود.  روش‌ها: این‌ پژوهش ‌یک ‌مطالعه ‌توصیفی- ‌تحلیلی ‌بود که‌ در سال 1390 انجام ‌شد. جامعه‌ آن متشکل از 280 نفر از پرستاران ‌کلیه ‌بیمارستان‌های ‌آموزشی ‌وابسته ‌به ‌دانشگاه‌ علوم پزشکی اصفهان بود. واحدهای مورد پژوهش ‌به‌ صورت تصادفی و روش ‌نمونه‌گیری‌ خوشه‌ای وارد مطالعه شدند. جهت ‌جمع‌آوری‌ داده‌ها ‌از یک‌ پرسش‌نامه ‌خود ساخته‌ سه بخشی استفاده ‌شد. تجزيه ‌و تحليل ‌داده‌ها با استفاده ‌از برنامه‌ آماري SPSS نسخه 18 (version 18, SPSS Inc., Chicago, IL) انجام‌ شد. یافته‌ها: بیشتر نمونه‌های مورد پژوهش زن (85 درصد) و سابقه کار کمتر از 5 سال (65 درصد) داشتند. در 20 درصد از واحدهای ‌مورد پژوهش ‌اشتباه ‌دارویی ‌اتفاق‌ افتاده‌ بود. میانگین ‌خطاهای ‌دارویی‌ پرستارا‌ن ‌در عرض‌ سه ‌ماه 11 مورد برای هر پرسنل برآورد گردید. این در حالی بود که میانگین گزارش ‌خطای ‌دارویی 5/1 مورد برای هر پرسنل در همین دوره زمانی بود. شایع‌ترین نوع اشتباهات ‌گزارش ‌شده شامل سرعت ‌انفوزیون‌ اشتباه با 19 درصد و دوز اشتباه با 12 درصد ‌بود. پرستاران تخمین زدند که 6/13 درصد از اشتباهات‌ دارویی طی فرایند خودگزارش‌دهی گزارش می‌شود. در این بین حیطه «ترس از عواقب گزارش‌دهی» به‌ عنوان شایع‌ترین علت گزارش ننمودن اشباهات دارویی عنوان ‌گردید. از هفت گویه موجود در حيطه ترس از پيامدهاي گزارش‌دهي عبارت «ترس از مسایل قضایی» بالاترين علت گزارش نكردن اشتباهات دارويي بود. نتیجه‌گیری: با توجه به اهمیت و تأثیر انکار نشدنی مبحث ایمنی بیماران، لزوم ایجاد یک سیستم کارامد گزارش‌دهی و ثبت خطا همراه با به حداقل رساندن موانع گزارش‌دهی جهت کاهش ‌اشباهات دارویی ضروری به ‌نظر ‌می‌رسد. همچنین پیشنهاد می‌شود که مدیران و مربیان پرستاری با هر چه غنی‌تر ساختن برنامه‌های آموزش ضمن خدمت، زمینه اعتلای دانش و مهارت پرستاران را در این حیطه میسر سازند. همچنین با توجه به این که بر اساس یافته‌های مطالعه حاضر ترس از پيامدهاي گزارش‌دهي یکی از علل مهم گزارش نكردن اشتباهات دارويي بود، بنابراین مدیران بايد يك واكنش مثبت در قبال گزارش‌دهي اشتباهات دارويي در پرستاران داشته باشند.

Title: به کارگيري مدل‌هاي بیزی، گاما-پوآسون، Lognormal و BYM در بررسي خطر نسبي خودکشي در شهرستان‌هاي استان ايلام

http://hsr.mui.ac.ir/index.php/jhsr/article/view/597

مقدمه: نقشه‌بندي بيماري‌ها شامل مجموعه‌اي از روش‌هاي آماري است که به تهيه نقشه‌هاي دقيق بر اساس برآوردهايي از ميزان‌هاي بروز، شيوع و مرگ و مير منجر مي‌شود. مدل‌هاي بیزی از مهم‌ترين مدل‌ها در اين حوزه به شمار مي‌آيد که اطلاعات پيشين تغييرات ميزان‌هاي بيماري در نقشه کلي و الگوي فضايي بيماري را در نظر مي‌گيرد. اين مدل‌ها دامنه گسترده‌اي را شامل مي‌شوند که هر کدام داراي فرمول‌بندي، ويژگي‌ها، نقاط ضعف و قوت مخصوص به خود هستند. در مطالعه حاضر به تشريح و مقايسه مدل‌هاي بیزی مهم و پرکاربرد گاما-پوآسون، Lognormal و BYM (Besag, York and Mollie) در بررسي خطر نسبي خودکشي در شهرستان‌هاي استان ايلام پرداخته شد. روش‌ها: پژوهش از نوع کاربردي- اکولوژيک بود. در اين پژوهش داده‌هاي خودکشي استان ايلام در سال 1386 و نيمه نخست سال 1387 مورد تحليل قرار گرفت. جهت تحليل داده‌ها از مدل‌هاي بیزی، گاما-پوآسون، Lognormal و BYM استفاده گرديد. برازش مدل‌ها به داد‌ه‌ها با استفاده از نرم‌افزار WinBUGS صورت گرفت. یافته‌ها: بر اساس برآوردهاي به دست آمده از برازش اين سه مدل، شهرستان‌هاي دره‌شهر و شيروان- چرداول به ترتيب بيشترين و کمترين خطر نسبي خودکشي را به خود اختصاص دادند. نتیجه‌گیری: با وجود تفاوت برآورد خطر نسبي در سه مدل، رتبه خطر نسبي شهرستان‌ها در هر سه مدل مشابه به دست آمد. شهرستان‌هاي استان ايلام از بيشترين تا کمترين خطر نسبي خودکشي به ترتيب دره‌شهر، ايلام، دهلران، ايوان، آبدانان، مهران، ملکشاهي و شيروان- چرداول بود. همچنين فواصل اطمينان و انحراف معيار به دست آمده از برازش اين سه مدل نيز تفاوت اندکي داشتند.

Title: ارزیابی شدت روشنایی سالن‌های مطالعه خوابگاه‌های دانشگاه علوم پزشکی اصفهان

http://hsr.mui.ac.ir/index.php/jhsr/article/view/1081

مقدمه: انسان بیشترین اطلاعات و آموخته‌های خود را از طریق سیستم بینایی به دست می‌آورد. سالن‌های مطالعه مکان‌هایی هستند که دانشجویان زمان زیادی را در آن‌ها به مطالعه می‌پردازند. روشنایی مناسب و توزیع بهینه آن می‌تواند خستگی چشم را به تأخیر اندازد و دقت و زمان مطالعه مفید را افزایش دهد. هدف این مطالعه بررسی میزان روشنایی سالن‌های مطالعه در خوابگاه‌های دانشگاه علوم پزشکی اصفهان بود. روش‌ها: مطالعه حاضر از نوع مقطعی بود که روی 24 سالن مطالعه در خوابگاه‌های دانشگاه علوم پزشکی اصفهان در سال 1389 انجام شد. پس از تهیه نقشه ساده از سالن‌ها و اندازه‌گیری ابعاد (طول، عرض و ارتفاع)، برای ارزیابی شدت روشنایی از روش شبکه‌ای استفاده شد. برای اندازه‌گیری شدت روشنایی از دستگاه نورسنج مدل HAGNER و در ارتفاع 60 سانتیمتر از زمین استفاده شد. داده‌های جمع‌آوری شده با استفاده از نرم‌افزار 16SPSS مورد تجزیه و تحلیل قرار گرفت. یافته‌ها: میانگین شدت روشنایی کلی، طبیعی و مصنوعی به ترتیب در 21، 9 و 2 سالن از کل سالن‌های مطالعه بیشتر از 300 لوکس استاندارد Illuminating engineering society of north america (IESNA) بود. میانگین نسبت مساحت پنجره به مساحت کف در خوابگاه دختران و پسران به ترتیب 12/0 و 14/0 بود. نتیجه‌گیری: روشنایی روز سالن‌های مطالعه با میانگین 567 لوکس در حد استاندارد بودند، اما روشنایی در شب به دلیل حذف منابع طبیعی و استفاده از منابع مصنوعی با میانگین 229 لوکس وضعیت مناسبی نداشتند.

Title: بررسي ميانگين مقادیر 10PM و 5/2PM موجود در هواي شهر خرم‌آباد

http://hsr.mui.ac.ir/index.php/jhsr/article/view/376

مقدمه: ذرات معلق یکی از مهم‌ترین آلاینده‌های هوا هستند و به ازای افزایش هر 10 میکروگرم بر متر مکعب از ذرات در هوا، میزان مرگ و میر 1 تا 3 درصد افزایش می‌یابد. ذرات معلق داراي بيشترين تنوع و پيچيدگي و از انتشار گسترده‌اي برخوردار هستند. اندازه، غلظت و ترکيب شيميايي ذرات معلق در هوا، مهم‌ترين ويژگي‌هاي آن‌ها است. مطالعه حاضر با هدف بررسی میزان آلودگی ذرات معلق (10PM و 5/2PM) در هوای شهر خرم‌آباد انجام گردید. مواد و روش‌ها: این مطالعه به صورت مقطعی و از نوع توصیفی بود که در فاصله زمانی یک سال (از فروردین تا اسفند 1389) انجام شد. نتایج با استفاده از آزمون آنالیز، واریانس و مقایسه زوجی شفه (Paired comparison) و آزمون One-sample t-test تجزیه و تحلیل شد. یافته‌ها: نتایج نشان داد که در طول دوره اندازه‌گیری، حداکثر غلظت 24 ساعته ذرات معلق مربوط به ایستگاه شمشیرآباد با میانگین غلظت 10PM و 5/2PM به ترتیب برابر 9/120 و 09/101 میکروگرم بر متر مکعب بوده است. مقایسه میانگین غلظت ذرات 10PM و 5/2PM در فصول مختلف نشان داد که بین میانگین غلظت ذرات فوق در فصول مختلف اختلاف معنی‌داری وجود داشته است (001/0 > P). نتیجه‌گیری: مقایسه سالانه میانگین غلظت 10PM و 5/2PM با مقدار استاندارد نشان می‌دهد که با وجود بالاتر بودن میانگین غلظت ذرات معلق در فصل تابستان، در مجموع میانگین غلظت ذرات فوق پایین‌تر از حد استاندارد بوده است.
